# Metabolic remodeling by the PD-L1 inhibitor BMS-202 significantly inhibits cell malignancy in human glioblastoma

**DOI:** 10.1038/s41419-024-06553-5

**Published:** 2024-03-04

**Authors:** Xueou Yang, Wenjun Wang, Tianhai Ji

**Affiliations:** 1 School of Medicine, Xiamen University, Xiamen, 361003 China; 2 College of Life Sciences, Guizhou Normal University, 550025 Guiyang, China; 3 Department of Pathology, Shanghai Ninth People’s Hospital, Shanghai Jiao Tong University School of Medicine, Shanghai, 200011 China

**Keywords:** Pharmacology, Cancer metabolism

## Abstract

Recently, crystallographic studies have demonstrated that BMS-202, a small-molecule compound characterized by a methoxy-1-pyridine chemical structure, exhibits a high affinity to PD-L1 dimerization. However, its roles and mechanisms in glioblastoma (GBM) remain unclear. The objective of this study is to investigate the antitumor activity of BMS-202 and its underlying mechanisms in GBM using multi-omics and bioinformatics techniques, along with a majority of in vitro and in vivo experiments, including CCK-8 assays, flow cytometry, co-immunoprecipitation, siRNA transfection, PCR, western blotting, cell migration/invasion assays and xenografts therapeutic assays. Our findings indicate that BMS-202 apparently inhibits the proliferation of GBM cells both in vitro and in vivo. Besides, it functionally blocks cell migration and invasion in vitro. Mechanistically, it reduces the expression of PD-L1 on the surface of GBM cells and interrupts the PD-L1-AKT-BCAT1 axis independent of mTOR signaling. Taken together, we conclude that BMS-202 is a promising therapeutic candidate for patients with GBM by remodeling their cell metabolism regimen, thus leading to better survival.

## Introduction

Glioblastoma (GBM) is the most common aggressive brain tumor of central nervous system, with an incidence of 5.8 per 100,000 persons. The five-year survival rate of GBM is <10%, with a medium survival time 14.6 months [1]. Currently, the diagnosis and treatment of high-grade gliomas still have a larger room for improvement, such as high cost, drug resistance, and poor ability to cross the blood–brain barrier [2, 3]. Small-molecule drugs, such as CA-170 [4], JQ1 [5], and BMS-202 [6], represent one such way to overcome the above dilemma.

Multiple factors and mechanisms play an important role in the development of GBM. Branched-chain amino acids (BCAAs) are the primary nitrogen source for synthesizing glutamate, which is a key substrate for catabolic and anabolic metabolism, and is indispensable for cell growth [7]. Therefore, treatments targeting BCAAs will likely improve tumor therapies [8, 9]. Programmed cell death ligand-1 (PD-L1) is commonly overexpressed in glioma cells and affects PI3K/AKT signaling pathways [10], which alternatively remodel the production of *BCAAs* [9]. However, it is still unclear whether the metabolism of *BCAA* is related to PD-L1 expression in GBM.

A previous study has reported that the blockade of PD-L1 could suppress the mTOR pathway and restrain glycolysis [11]. Additionally, PD-L1 has been found to structurally interact with some small molecules synthesized by Bristol Myers Squibb (BMS) Ltd., one of which was a co-crystal complex structure of the small-molecule BMS-202, which interacted with human PD-L1 to induce the formation of PD-L1 homodimers [12, 13]. However, to our present knowledge, no studies have examined the binding of BMS-202 with PD-L1 in GBM cells, and it is still unclear whether it has effects on remodeling the cell metabolism, particularly BCAAs, to prevent their growth. Therefore, in the study, our major goals are to investigate whether and how BMS-202 remodels the BCAAs metabolism by affecting the expression of PD-L1 to block cell proliferation in GBM.

## Results

### BMS-202 effectively inhibits the malignant phenotypes by interfering with the expression of PD-L1 in GBM cells

**Fig. 1:**
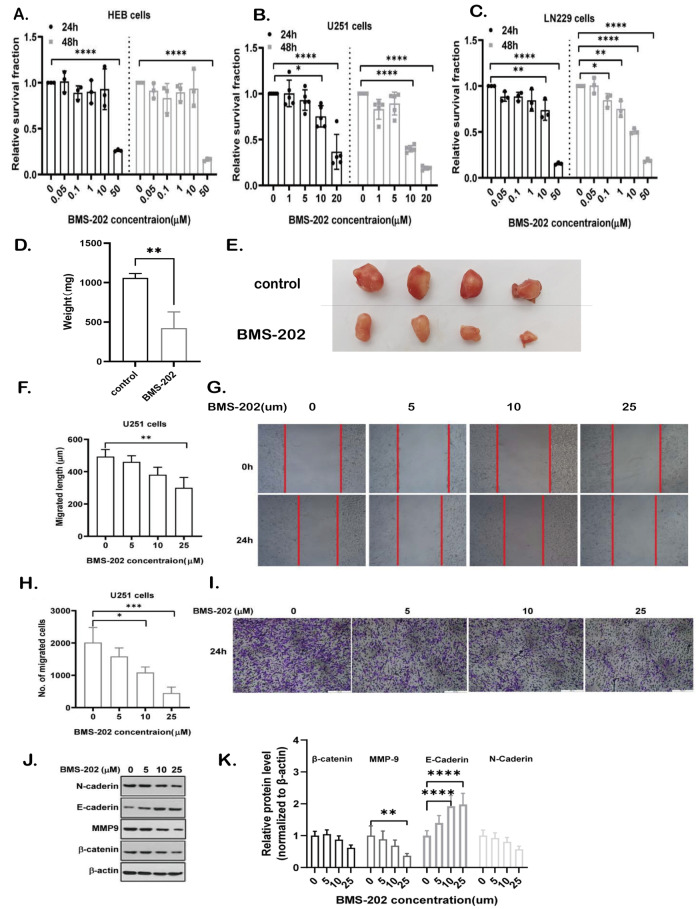
BMS-202 inhibits cell malignancy in GBM cell lines. **A**–**C** Cell viability analysis of HEB (**A**), U251 (**B**), and LN229 (**C**) cells treated with indicated concentrations of BMS-202 for 24 h and 48 h, respectively. Data from replicates were shown as mean ± s.d. and statistically analyzed using ANOVA test, which was shown as barplots with individual points. **D**, **E** The tumor weight (mg) (**D**) and subcutaneous tumors (**E**) from U251-bearing nude mice treated with vehicle (control) or 20 mg/kg BMS-202. Data were shown as mean ± s.d. and statistically analyzed using Students’ *t* test. **F**, **G** Cell migration analysis of U251 cells treated with indicated concentrations of BMS-202 for 24 h. Data from replicates were shown as mean ± s.d. and statistically analyzed using ANOVA test. **F** Representative data were shown in **G**. **H**, **I** Cell invasion analysis of U251 cells treated with indicated concentrations of BMS-202 for 24 h. Data from replicates were shown as mean ± s.d. and statistically analyzed using ANOVA test (**H**). Representative data were shown in **I**. **J**, **K** Western blotting assays of cell migration and invasion biomarkers with indicated antibodies (**J**), the gray values of which were shown using barplots (**K**). Data from replicates were shown as mean ± s.d. and statistically analyzed using ANOVA test, *p < 0.05, **p < 0.01, ***p < 0.001.

We first assessed the antitumor activity of BMS-202 in GBM cells using CCK-8 assays. The results showed that after 24 h of treatment with BMS-202 (10 μM), the cell viability of U251 and LN229 cells was significantly reduced (*p* < 0.01), while that of the normal glial cell line (HEB) was not significantly affected (Fig. 1A–C). Moreover, data from tumor-bearing nude-mice xenografts supported that BMS-202 significantly inhibited the growth of U251 cells in vivo (Fig. 1D, E). Above all, these in vivo and in vitro data demonstrated that BMS-202 significantly inhibited the growth of GBM cells without affecting normal glial cells, implicating a safe therapeutic window for its antitumor application in GBM.

Next, we further evaluated its roles in cell migration and invasion. Interestingly, BMS-202 significantly suppressed the cell migration at 25 μM (Fig. 1F, G) and inhibited the cell invasion at 10 μM (Fig. 1H, I). Likewise, we further detected the expression of key factors involved in epithelial-mesenchymal transition (EMT) process, such as N-cadherin, E-cadherin, β-Catenin, and MMP-9 [14, 15], western blotting results of which showed that it apparently increased the expression of E-cadherin, a negative factor in EMT, and reduced the expression of MMP-9, a positive regulator in EMT, at the same time in a concentration-dependent manner (Fig. 1J, K, *p* < 0.01), while there were no significant difference in the expression of β-Catenin and N-caderin in U251 cells (Fig. 1J, K).

We further investigate the inhibitory activity of BMS-202 on PD-L1 expression either on the surface or in the cytoplasm of GBM cells. Firstly, we tested the expression of PD-L1 on the surface of U251 cells in response to 24 h treatment of 10 μM BMS-202 using flow cytometry. As the expression of PD-L1 is weak enough, the IFN-γ (500 IU/mL) was employed to further stimulate its expression [16, 17]. Interestingly, the expression of PD-L1 on the surface of U251 cells was significantly increased in response to IFN-γ stimulation (p < 0.05), while under the same conditions as mentioned above, the increase was not significant when treated with BMS-202 (Figure S1A). Meanwhile, the mRNA and protein levels of PD-L1 in U251 cells treated with BMS-202 were further examined using RT-PCR and western blotting assays, respectively. Unexpectedly, the results showed that it had no significant impacts on the mRNA and protein levels of PD-L1 (Fig. S1B, C). However, it had a significant inhibition on the cell migration in U251 cells transfected with siRNAs targeting PD-L1 (Fig. S1D, Table S3), the knockdown efficiency of which was confirmed by western blotting and RT-PCR assays (Fig. S1E).

Above all, the results indicated that BMS-202 notably suppressed GBM cell proliferation, invasion, and migration by affecting PD-L1 activity on the cell surface, rather than affecting its transcriptional and translational levels.

### BMS-202 remodels the metabolic landscape by reducing the production of L-isoleucine in GBM cells

**Fig. 2:**
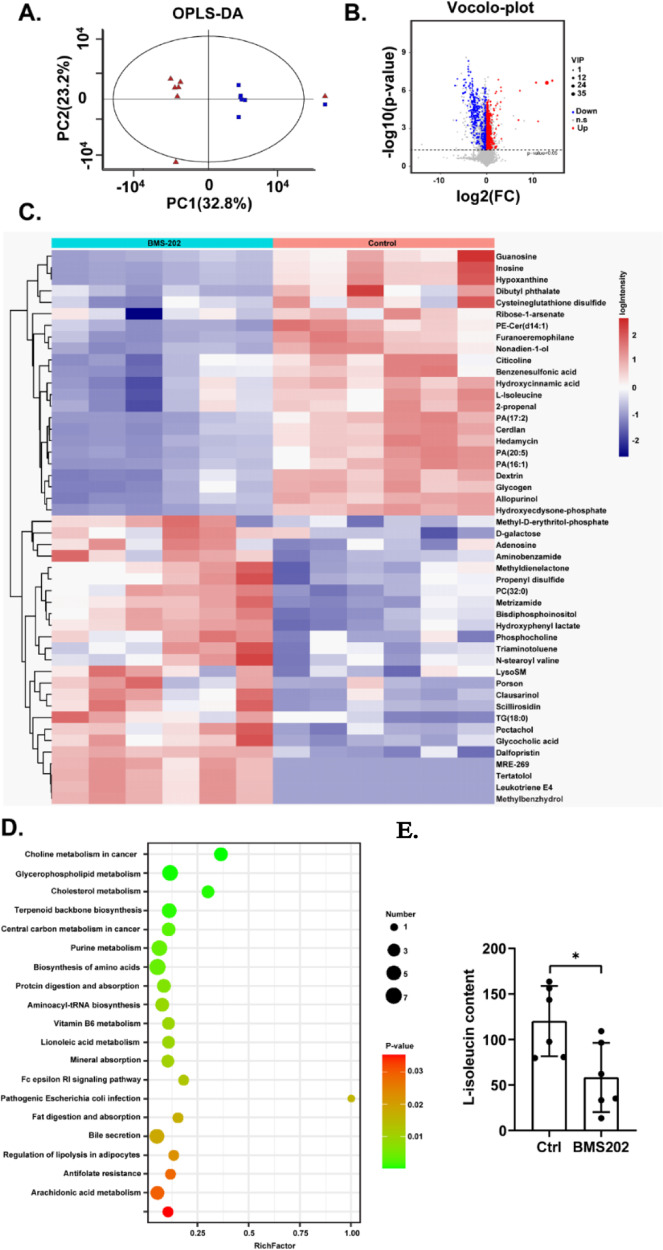
BMS-202 remodels the metabolic landscape by reducing the content of L-isoleucine in GBM cells. **A** OPLS-DA score plots between the control and BMS-202 groups. **B** Volcano plots showing the differentially distributed metabolites in BMS-202 group, taking the vehicle group as a control. red represents upregulated metabolites, and blue represents downregulated metabolites. **C** Clustering heatmap of typical metabolites; colors indicate the intensity level of the metabolites, with red representing a high level, and blue representing a low level. **D** GSEA enrichment of the pathways significantly participated by the above differential metabolites. **E** Barplots with individual points showing results from Enzyme-linked immunosorbent assay that BMS-202 inhibited the production of L-isoleucine in U251 cells. *p < 0.05.

We first investigated how BMS-202 affected GBM cells using transcriptomic sequencing technology. As shown in Figure S2A, the 1796 differentially expressed genes (DEG) (FC > 2, *P* < 0.05) in U251 cells were visualized using volcano plots, with log10 (*Q* value) as the ordinate and log2 (drug/control, FC) as the abscissa. FC represents the ratio between the gene expression levels in the treatment group and the control group samples. According to the significant DEG selection criteria that |log2(drug/control) |>1 and Q-value < 0.05, a total of 42 genes were found in the BMS-202 treated group when compared to the control group, with 21 upregulated genes and 21 downregulated genes, respectively. Subsequently, we listed the detailed information of the mentioned 42 genes in Table S1 for convenient analysis. Furthermore, we performed GO biological function and KEGG pathway analysis using KOBAS (http://kobas.cbi.pku.edu.cn) and Omic (https://www.omicshare.com), obtaining 1203 significantly correlated GO terms and 57 KEGG pathways. Among the 1203 GO terms, metabolic progress was one of the top-ranked biological progress using GO annotation and KEGG pathway analyses (Figure S2B). Additionally, the top 25 KEGG pathways were ordered in a descending sequence according to the p-values, and the metabolism pathways, including nitrogen metabolism, ether lipid metabolism, and the amino sugar and nucleotide sugar metabolism, were identified as the main KEGG pathways (Figure S2C), implicating that differential genes in response to BMS-202 were mainly enriched in the metabolic progress, which included approximately 162 genes. The detailed metabolic process is shown in Figure S2D. Next, we further examined the metabolic alterations in GBM cells using metabolomics technology. Principal component analysis (PCA) was performed to determine those significantly different metabolic patterns between the BMS-202 treated group and the control group. Afterwards, the orthogonal partial least squares-discriminant analysis (OPLS-DA) was further used to maximize the difference between groups (Fig. 2A). The parameters R2 and Q2 in the permutation test were lower than the original ones, and the *Y* axis intercept for Q2 was <0, suggesting that the OPLS-DA model was stable and reliable, without overfitting, and was therefore suitable for subsequent analysis. Taking the Variable important in projection (VIP > 1) and *p* value (*p* < 0.05) as the criteria in the OPLS-DA model, 273 different metabolites were discovered (Fig. 2B) and enriched by the heatmap analysis (Fig. 2C). Alignment was performed among the top 50 VIP metabolites through the PubChem and MetaboLights databases. Ultimately, six potential metabolic biomarkers (Table 1) were identified, which were significantly downregulated in GBM cells when compared to the control group (FC < 1). Afterwards, the differential metabolites were enriched by the KEGG pathway assays, results of which showed that these metabolites mainly participated in choline metabolism, cholesterol metabolism, and amino-acid metabolism (*p* < 0.01) (Fig. 2D). Previous reports have shown a close relationship among arginine, glutamine, and BCAA metabolism in cancer cells [18, 19], thus, we chose L-isoleucine, one of the BCAA metabolites, as our main focus. Interestingly, the results from ELISA assays showed that the content of L-isoleucine was dramatically decreased in BMS-202 treated group when compared to the control group (Fig. 2E, *p* < 0.05).

**Table 1:** List of potential metabolites identified in the study.

No.	Metabolites	Formula	MW	KEGG No.	FC
1	Inosine	C10H12N4O5	268.23	C00294	0.19
2	Allopurinol	C5H4N4O	136.1	DG00779	0.25
3	L-Isoleucine	C6H13NO2	131.17	C00407	0.77
4	Citicoline	C14H26N4O11P2	488.32	C00307	0.52
5	Guanosine	C10H13N5O5	283.24	C00387	0.23
6	Hypoxanthine	C5H4N4O	136.11	C00262	0.20

*MW* molecular weight, *KEGG* Kyoto Encyclopedia of Genes and Genomes, *FC* fold change.

### BMS-202 activates Akt by attenuating the interaction between PD-L1 and- Akt

**Fig. 3:**
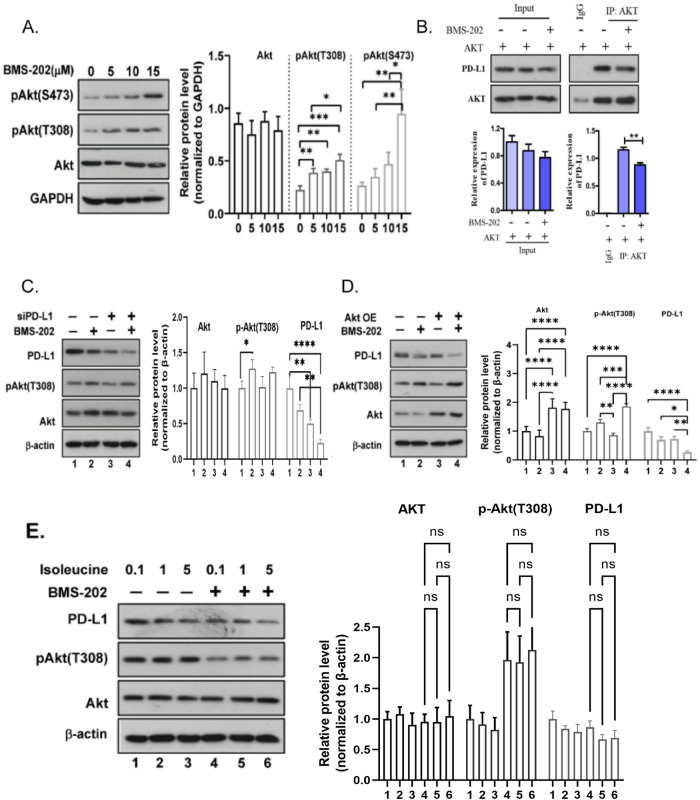
BMS-202 activates AKT by attenuating the interaction between PD-L1 and AKT. **A** Western blotting results showing the activity of AKT indicated by P-AKT (T308) and P-AKT (S473) in U251 cells treated with different concentrations of BMS-202. The gray bands from replicates were shown as mean ±s.d. and statistically analyzed using ANOVA test. **B** Co-immunoprecipitation results showing the interruption of BMS-202 on the interactions between PD-L1 and AKT in U251 cells. **C**, **D** Western blotting results showing the expression of indicated proteins in U251 cells transfected with or without siPD-L1-2 (**C**) or AKT overexpressing plasmids (**D**) in response to BMS-202 treatment. **E** Western blotting results showing the expression of indicated proteins in U251 cells treated with indicated concentrations of Isoleucine, together with or without BMS-202. *p < 0.05, **p < 0.01, ***p < 0.001, ****p < 0.0001. Data from replicates are all shown as the mean ± s.d. and were statistically analyzed using ANOVA.

With the aim to investigate the possible mechanisms by which BMS-202 down-regulates the L-isoleucine content while inhibiting the effects of PD-L1, we performed western blotting to further verify the relationship between PD-L1 and Akt. Akt is generally phosphorylated at the 308 site of threonine (T308) in the activation loop and the 473 sites of serine (S473) in the hydrophobic motif of its C-terminal tail, both of which are markers for Akt activity [20]. Initially, the expression of p-Akt (T308) and p-Akt (S473) was detected in U251 cells, and the results revealed that both p-Akt (T308) and p-Akt (S473) were significantly increased in response to BMS-202 treatment (Fig. 3A, *p* < 0.01). Lysates from U251 cells were then subjected to AKT immunoprecipitation. The results showed that an interaction between PD-L1 and AKT in U251 cells was significantly attenuated by BMS-202 (Fig. 3B, *p* < 0.01). Next, the association between PD-L1 and AKT was studied by transfecting siRNA targeting PD-L1 and AKT overexpression, respectively. Interestingly, BMS-202 with PD-L1 knockdown was no longer effective at upregulating p-Akt (T308) expression (Fig. 3C), while the expression of PD-L1 was further reduced in AKT overexpressing cells treated with BMS-202 (Fig. 3D). Overall, our data indicated that BMS-202 indeed affected AKT protein expression by altering its interaction with PD-L1 in GBM. According to reports [21] BCAA may enhance the drug’s anti-cancer potential. However, L-isoleucine showed little effect on improving BMS-202 roles in GBM cells (Fig. 3E).

### BMS-202 has no significant effects on mTOR signaling cascade activity

**Fig. 4:**
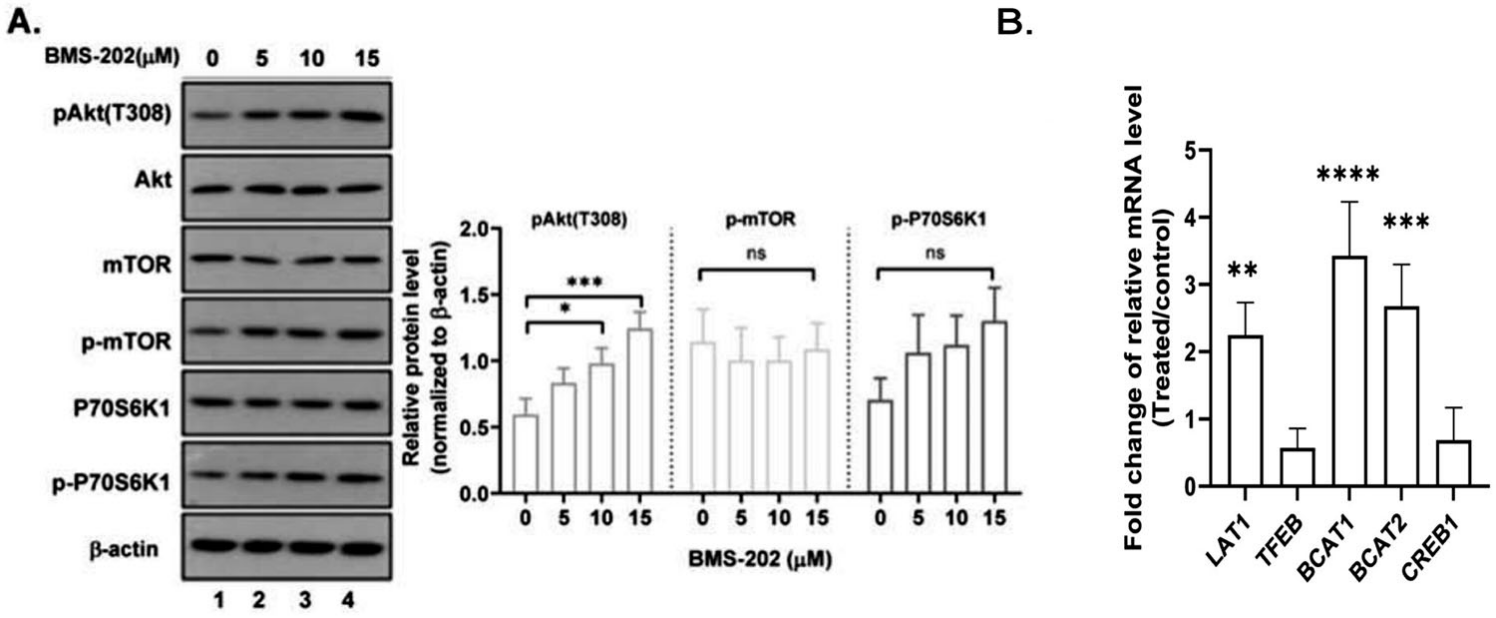
BMS-202 has no significant role in regulating the Akt downstream mTOR signaling. **A** Western blotting results show the expression of indicated proteins in U251 cells in response to different concentrations of BMS-202. **B** RT-PCR results showing the relative expression of indicated genes in U251 cells treated with or without 10 μM BMS-202. *p < 0.05, **p < 0.01. Data from replicates are all shown as the mean ± s.d. and were statistically analyzed using ANOVA.

The mechanistic target of rapamycin kinase (mTOR) plays a central role in regulating cell growth and proliferation [20]. To study whether BMS-202 treatment affects the Akt-mTOR pathway by regulating L-isoleucine metabolism, representative p-mTOR, mTOR, p-S6K, and S6K proteins were detected using western blotting assays, results of which determined that the expression of the above-mentioned proteins was not significantly altered in response to BMS-202 treatment (Fig. 4A). Previous reports have shown some Akt-regulatory genes, including transcription factor EB (TFEB), cAMP response element binding protein 1 (cAMP [22], L-type amino-acid transporter (LAT1) [23], branched-chain amino-acid transaminase 1 (BCAT1), and branched-chain amino-acid transaminase 2 (BCAT2). Consistently, our qPCR results showed that the relative expression of *LAT1*, *BCAT1*, and *BCAT2* in BMS-202 group was significantly higher than that in the control group (Fig. 4B, *p* < 0.05). Overall, the results showed that changes in PD-L1 and Akt protein expression in BMS-202-treated U251 cells involved alterations of BCAT1/2 but not the mTOR signaling pathway.

### BMS-202 positively regulates the expression of BCAT1 via Akt activity

**Fig. 5:**
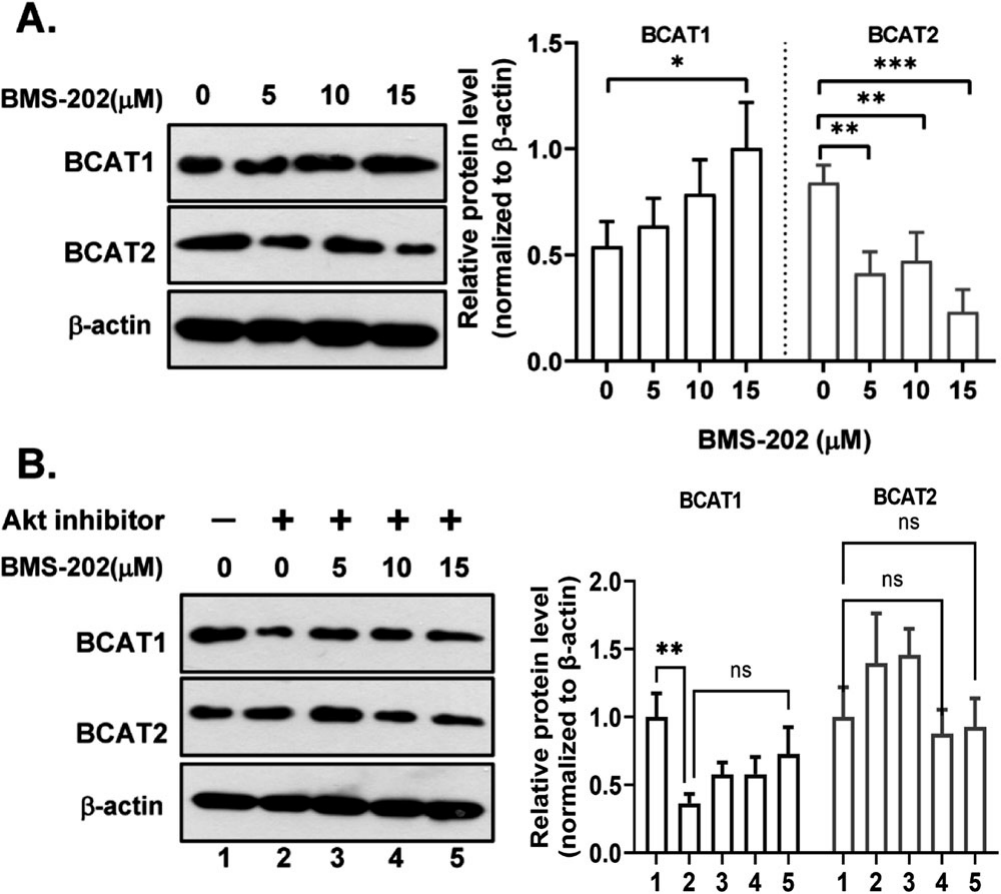
Effects of BMS-202 on the expression of BCAT1/2 in GBM cells. **A** Western blotting results showing the expression of indicated proteins in U251 cells treated with indicated concentrations of BMS-202. **B** Western blotting results show the expression of indicated proteins in U251 cells treated with Akt inhibitor LY294002, together with or without BMS-202. *p < 0.05, **p < 0.01. Data from replicates are all shown as the mean ± s.d. and were statistically analyzed using ANOVA.

Subsequently, the protein levels of both BCAT1 and BCAT2 were further examined in U251 cells treated with or without BMS-202 using western blotting assays. As shown in Fig. 5A, the expression of BCAT1 was highly increased in 15 μM BMS-202 treated U251 cells, which matched their above mRNA results. However, the BCAT2 expression was significantly inhibited by BMS-202 in a concentration-dependent manner, which was contrary to its mRNA change, suggesting a complex role of BMS-202 in regulating BCAT1 and BCAT2 proteins.

To further investigate the direct role of BMS-202 in manipulating the expression of BCAT1/2, we proposed that the Akt inhibitor LY294002 might reverse the changes in BMS-202-treated GBM cells. The results from western blotting assays showed that LY294002 significantly suppressed the expression of BCAT1 in GBM cells, which successfully blocked its upregulation induced by BMS-202 (Fig. 5B). However, there was no significant change in BCAT2 in these treated groups. Taken together, these results showed that BMS-202 positively regulated the expression of BCAT1 by activating Akt to inhibit tumor progression in GBM.

### BMS-202 metabolic genes related to the clinical consequences in patients with high-grade glioma

**Fig. 6:**
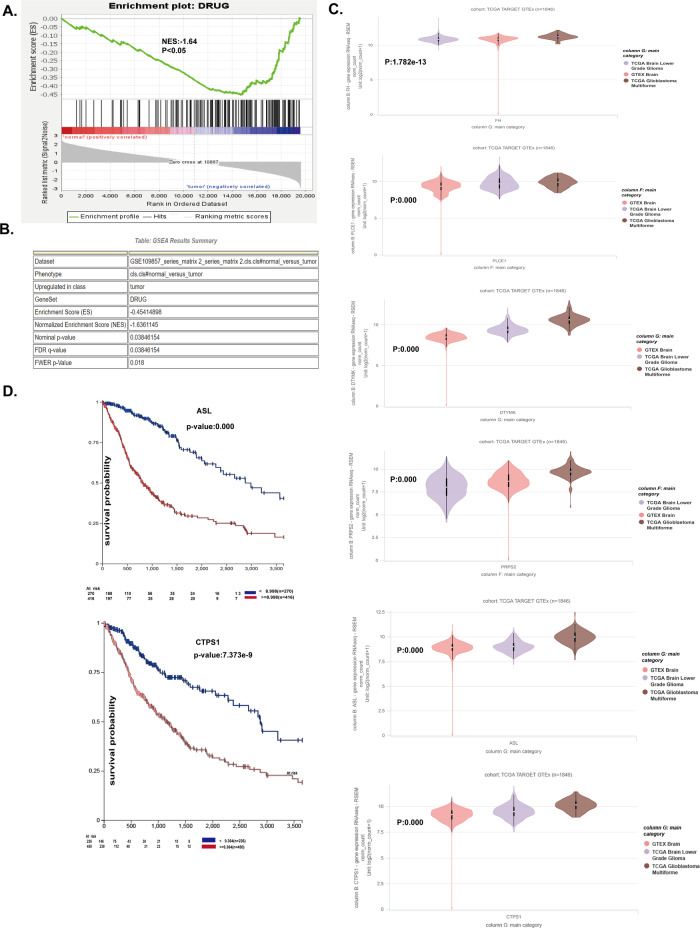
BMS-202 metabolic genes related to the clinical consequences in patients with glioma. **A** Analysis of GSEA between drug-metabolic genes and glioma (NES: –1.64, *p* < 0.05. **B** Different expression of BMS-202 metabolic-related genes between normal and glioma group. **C** Different expression of Hub genes (pLCE1, PRPS2, FH, ASL, DTYMK, and CTPS1) in different grades of glioma. **D** BMS-202 metabolism-related hub genes are closely associated with glioma poor prognosis. Taking the normal group as a control, data were shown as mean ± s.d. *t* test was used to determine the statistical significance.

To analyze the value of BMS-202 in terms of clinical application, we further predicted the difference in the expression of BMS-202 metabolic genes between normal and high-grade glioma groups. As shown in Fig. 6A, B, these genes were significantly different in tumor and normal tissues, and showed a strong inverse relationship in patients with tumor, in that the upregulated genes were enriched for patients with tumor and the downregulated genes were enriched for the normal group (NES: –1.64, *p* < 0.05). The core-enriched genes with gene set enrichment analysis (GSEA) contained 73 metabolic genes, the details of which were shown in Table S2. Of these, 37 genes were downregulated and 36 genes were upregulated. Accordingly, the top 15 downregulated genes were selected for downstream analysis. Furthermore, we found that six downregulated genes, including pLCE1, PRPS2, FH, ASL, DTYMK, and CTPS1 were significantly increased in high-grade glioma tissues (Fig. 6C, *p* < 0.001). Besides, patients with low expression of above six genes showed better survival rates than those with high expression (Fig. 6D and S3A–D. *p* < 0.001). Above all, we can safely conclude that BMS-202 may favor the metabolic genes of patients with high-grade glioma toward good aspect and may prolong patients’ survival time.

## Discussion

PD-L1 is overexpressed on the surface of glioma cells where it forms the PD-1/PD-L1 complex, which is one of the most important causes for glioma immune evasion [24, 25]. Small-molecule inhibitors targeting PD-1/PD-L1 are one of the main methods of tumor immunotherapy due to their low cost, low toxicity, and synergistic effect with biologic immunosuppressants. Previous studies have suggested that BMS-202 interacts with the protein PD-L1 [12, 13] to show similar antitumor efficiency [26, 27]. However, no further information has been reported regarding the mechanism of BMS-202 in glioma tumors. Therefore, the exploration of its biological functions and mechanisms in GBM cells is of great importance.

In the current study, we first verified that BMS-202 could upregulate BCAT1 in the cytosol to promote L-isoleucine decomposition, which inhibited the malignant phenotype of GBM cells. We also revealed that BMS-202 attenuated PD-L1-bound Akt by downregulating PD-L1 to activate Akt signaling. Moreover, we propose that BMS-202 could downregulate some metabolic genes and prolong the survival of patients with glioma.

Programmed cell death ligand-1 (PD-L1) is distributed across the cytosol and surface of glioma cells. Here, we reported that enhancing PD-L1 expression on the tumor cell surface by IFN-γ showed good performance for detecting the effects of BMS-202 [28], and validated this interaction between BMS-202 and PD-L1 in glioma cells [12, 13]. The effects of BMS-202 were not obvious at the protein and mRNA levels in glioma cells; therefore, we speculate that the effects of BMS-202 treatment occur predominantly on the glioma cell surface and that changes in glioma cells are derived from alterations in the PD-L1 structure, which is consistent with the findings from previous studies [12, 13].

AKT, also known as AKT serine/threonine kinase 1, is involved in the regulation of many cellular processes, including survival, proliferation, growth, and glycogen metabolism, and as such, correlates with the development and progression of many diseases. PD-L1 overexpression in glioma cells can cause changes in six signal pathways, including PI3K-AKT [29]. Importantly, our results further confirmed that BMS-202 affected AKT expression and demonstrated that it could attenuate the binding of PD-L1 with Akt by modulating the structure of PD-L1, rather than affecting its mRNA and protein levels. Additionally, BMS-202 also activated the AKT signaling by inducing its phosphorylation, indicated by p-Akt (S473) and p-Akt (T308). However, whether AKT trans-locates from cytosol to cellular membrane still needs further studies.

Additionally, metabolic reprogramming events are mainly manifested by changes in the genes responsible for the rate-limiting steps [30]. BCAT1/2 is a typical rate-limiting step for L-isoleucine decomposition, which contributes to BCAA metabolism. Here, we determined amino-acid metabolic alterations in GBM cells using multi-omics approaches [31]. Thus, L-isoleucine was capable of driving tumor metabolic reprogramming in GBM cells. Another important finding in our study was that BMS-202 significantly induced changes in the expression of BCAT1/2 protein and mRNA in GBM cells, confirming its role in remodeling the metabolic landscape. Taken together, these results suggest that BMS-202 is critical in reprogramming the cell metabolism by modulating the PD-L1/AKT/BCAT1 signaling pathways, which shed novel insights into previous BMS-202-associated signaling pathways [32, 33].

Additionally, for the first time, we report that BMS-202 metabolic genes at the metabolism level are associated with clinical consequences in patients with glioma, including the duration of survival times. Therefore, our results present interesting clinical evidence pertaining to the value of BMS-202 in the treatment of glioma. However, whether BMS-202 can be described to extend lifespan and delay metabolism still requires further validation. In summary, BMS-202 can inhibit PD-L1 and exert metabolic remodeling in glioma cells as a tumor suppressor by inhibiting the tumor malignant phenotype.

## Material and methods

### Chemicals and reagents

BMS-202 (BCP16068, purity: 99.3%) was purchased from BioChemPartner (Shanghai, China)**;** primary and secondary antibodies were from Cell Signaling (Boston, USA), IFN-γ (300-02-100) was from PeproTech (Rocky Hill, USA); and anti-human CD274 (PD-L1, 557924) was from Becton, Dickinson and Company (BD, State of New Jersey, USA).

### Cell lines and culture

U251, LN229, and HEB cells were kindly acquired from XiaMen Chenggong Hospital Laboratory and cultured in Dulbecco’s modified Eagle medium (DMEM)-high glucose medium (Hyclone, USA) containing 10% fetal calf serum (Gibco, USA), 1% penicillin, and 1% streptomycin (Hyclone, USA). All cell lines were maintained at 37 °C in a humidified atmosphere containing 5% CO_2_^.^

### Plasmids and transfection

The siRNAs targeting PD-L1 or control (Table S3), together with plasmids expressing the full length of AKT1 with primers (Table S3) between EcoR I and Xho I sites on pCMV-Myc vector were purchased from Thermo Fisher Scientific Inc. (Waltham, USA) and Sino Biological Inc. (Beijing, China), respectively. Transfection was performed when the U251 cells reached a confluency of 70%. The plasmids were added to Opti-MEM medium (100 μl) for 5 min, before adding 2 μl Lipo2000 reagent and mixing for 20 min at room temperature. Next, the cell medium supernatant was removed, and the serum-free medium (2 ml) with the above mixture was added to the cell culture for 6 h at 37 °C. Finally, cells were incubated for 48 h in the new DMEM and detected using western blot and RT-PCR.

### Drug constitution

To prepare BMS-202, it was first dissolved in dimethyl sulfoxide (DMSO) at a dose of 3.9 mg/100 μl, before taking 20 μl and solubilizing in 5 ml DMEM containing 0.5% fetal bovine serum (FBS). Finally, DMEM medium with 0.5% FBS was added and diluted to achieve a drug concentration of 10 μM.

### Cell proliferation assay

For the cell proliferation assay, 1–1.5 × 10^3^ cells/well were seeded in 96-well plates and cultured for 24 h and 48 h. The control group included DMEM without BMS-202; the blank group was without any cells or culture media; the vehicle included BMS-202 solution without cells. CCK-8 assays were conducted using a CCK-8 kit (MCE, USA), with absorbance detection at 490 nm. Viability (100%) = (*A*–*A*_vehicle_)/(*A*_control_–*A*_blank_) × 100%.

### Scratch and invasion assays

For the scratch assay, U251 cells were seeded in 6-well plates (Corning, NY, USA) *and* cultured until 90*%* confluence, following which 10-μ*l* pipette tips were used to scratch the cells. After washing the cells in phosphate-buffered saline (PBS) (Thermo Fisher, USA), serum-free DMEM and BMS-202 were added. A series of images were taken over a 24-h period and analyzed by Image J.

For the invasion assay, U251 cells (2 × 10^4^*)* suspended in 100 μl serum-free DMEM were incubated in transwell chambers (Corning, NY, USA) with 20% Matrigel (BD, Franklin Lakes, NJ, USA). After 24 h of BMS-202 treatment, the cells in the bottom of the chamber were fixed in 4% paraformaldehyde and stained with 0.4% crystal violet.

### Flow cytometry assay

After 24 h of BMS-202 treatment in combination with 500 IU/ml IFN-γ stimulation (peprotech*, USA)*, U251 cells (1.5 × 10^*5*^) in six-well plates were trypsinized and centrifuged for 5 min at 4 °C. Then, after washing the cells in Stain Buffer BSA (BD Pharmingen, USA), 20 μl of anti-PD-L1 (BD Pharmingen, USA) was added with an equal volume of bovine serum albumin (BSA) and incubated on ice for 30 min in the dark. Subsequently, the cells were filtered through a 250-μm nylon mesh, washed twice with *BSA*, and analyzed *by* flow cytometry *(*BD FACS Aria III cell sorter, USA) within 1 h.

### Quantitative real-time (RT)-PCR

Total RNA extracted using TRIzol reagent (Aidlab, Beijing, # RN03) was reverse-transcribed using a PrimeScript TM RT Reagent Kit (Takara, Japan, #FSQ-101). RT-PCR was performed using Bestar® qPCR Master Mix (DBI, German, #2043) following the manufacturer’s instructions. The relative gene expression was normalized to β-actin. The sequence of primers used in the study is summarized in Table S3.

### Western blotting

Western blotting was performed as previously described [16]. Briefly, U251 cell lysates were lysed in RIPA buffer and the concentration was measured by the BCA method. Subsequently, the protein lysates were subjected to standard SDS*-*PAGE and transferred onto PVDF membranes *(Millipore)*. After blocking with 5% milk in TBST buffer, the membranes were incubated with the primary antibodies at 4 °C overnight. All bands were exposed to enhanced chemiluminescence (ECL) (Advansta, USA) on a UVP BioSpectrum imaging system, and the relative levels of proteins were calculated by normalizing to the internal control β‐actin. All the antibodies used in the study are summarized in Table S4.

### Co-immunoprecipitation (Co-IP) assay

Co-IP was performed using the Pierce co*-*IP kit according the manufacturer’s protocol. Briefly, after 24 h of BMS-202 treatment, the U251 cells grown were harvested from 6-well plates in 500 μl of IP lysis buffer (Thermo Fisher, USA, 87788) with a Protease inhibitor Cocktail (1:100, Sigma-Aldrich). Then, the lysate was incubated with 5 μl of antibody against AKT and 120 µl of Protein A/G magnetic beads at 4 °C overnight on a rocking platform. Upon precipitation, proteins were separated by centrifugation at 3000 rpm for 3 min at 4 °C and boiled in 30 μl loading buffer for analysis of PD-L1 expression by western blotting.

### Enzyme-linked immunosorbent (ELISA)

ELISA was performed as previously described [14]. The Jianglai’s Human L-isoleucine (Shanghai, China) in vitro ELISA kit (JL47019) was used to detect the concentration of L-isoleucine in the cell supernatant. Briefly, 100 μl working solutions for ELISA were added into each well and incubated at 37 °C for 30 min. After washing five times, the TMB solution was added and incubated for 15 min. Samples were analyzed in triplicate using the Varioskan Flash (Thermo Shandon), and the absorbance was measured at 450 nm.

### Transcriptomics analysis

After setting up the control and medication groups, 2 × 10^5^ U251 cells in triplicate for each group were harvested, washed three times with PBS, and dissolved in 1 ml TRIzol reagent for 5 min, then 200 μl precooled chloroform was added for RNA extraction. We contracted Shenzhen Huada Gene Technology Co., Ltd. to perform transcriptomic analyses. Total RNA quality and integrity evaluation were performed using nondenaturing agarose gels. Then, after oligo (dT) magnetic beads-assisted enrichment, mRNA fragmentation was conducted. The synthesis of double-strand cDNA was followed by adaptor ligation and end-repair. PCR amplification was performed to generate a circular DNA library. The nucleic acids were pelleted after quality checking and then sequenced by combinatorial probe-anchor synthesis (cPAS). SOAPnuke filtering software was used to obtain accurate sequencing data from the raw data. Then, the clean reads were aligned with a reference genome sequence using HISAT. A *Q* value of 0.05 and |log_2_ (drug/control)|>1 were used as thresholds for significantly DEG. In DEGs, enrichment analyses of GO and KEGG pathways were performed using KOBAS 3.0 (http://kobas.cbi.pku.edu.cn/) and cytoscape3.8.2 (https://cytoscape.org/), respectively.

### Ultra-high liquid chromatography-tandem mass spectrometry assay (UPLC-MS/MS)

U251 cells (2 × 10^6^) seeded in six-well plates were collected in six replicates, mixed with 1 ml chilled methanol/water (4:1, v-v) and 200 μl chloroform, and were further ultrasonically broken in an ice bath for 3 min. After adding internal standards, the solution was sonicated for 20 min and deproteinized by centrifugation at 4 °C (13,000 rpm, 10 min). Finally, 1 ml of the supernatant was evaporated, reconstructed with 200 μL methanol/water (1:4, v-v), further incubated for 2 h at −20 °C, and centrifugated prior to analysis. Metabolic extracts were analyzed using a Nexera UPLC-MS/MS system (Shimadzu, Japan). Chromatographic separation was conducted on a Waters ACQUITY UPLC HSS T3 (100 mm × 2.1 mm, 1.8 μm) at a flow rate of 0.35 mL/min, with acetonitrile (containing 0.1% formic acid) and water (containing 0.1% formic acid). The electrospray ionization (ESI) operation of the Waters Xevo TQD mass spectrometer (Waters Milford, USA) was used in the positive (ESI+) and negative (ESI–) ion modes; the negative and positive modes were 3.0 and 3.5 kV, respectively, and the drying gas (N2) flow rate was 10.0 L/min 320 °C, Data were recorded from 50 to 1000 m/z.

### Multivariate pattern analysis

Multivariate analysis (MVA) was performed using SIMCA-P software v14.0+ from Umetrics (Umea, Sweden) for PCA, projection to latent structures-discriminant analysis (PLS-DA), and orthogonal projection to latent structures-discriminant analysis (OPLS-DA) to identify the differences in metabolites between groups. The threshold for a significant difference was VIP > 1, *t* test *p* < 0.05, and fold change (FC) > 1.2.

### In vivo therapeutic tumor-bearing xenografts with BMS-202

According to our previous studies [18], 5 × 10^6^ U251 cells were mixed with matrigel and subcutaneously injected into the male NOD/SCID nude mice, aged 4–6 weeks (*n* = 8). When the tumor volume (0.5 × length × width^2^) reached 100 mm^3^, the mice were randomly divided into the control group, treated with vehicle, and the BMS-202 group, intraperitoneally injected with 20 mg/kg BMS-202, twice per week. The therapeutic process was stopped until the tumor volumes in the control group reached the ethically approved maximum volume 2000 mm^3^. This animal study was conducted according to guidelines approved by the Laboratory Animal Ethics Committee of Shanghai Ninth People’s Hospital, Shanghai Jiao Tong University School of Medicine (protocol no. SH9H-2023-A890-SB).

### BMS-202 metabolism-related genes in clinical studies

To further assess the value of BMS-202, hub genes based on metabolism that can influence glioma and its treatment in clinical situations were studied using the GEO dataset, GSEA dataset, and UCSC dataset. First, we downloaded GSE109857 microarray data from GEO, comprising 225 samples. Next, we submitted BMS-202 metabolism-related genes to GSEA4.3.2 software to determine differences between the normal and glioma groups. We also analyzed the association between these genes and the survival of patients with UCSC and a *p* < 0.05.

### Statistical analyses

The results are shown as the mean ± standard deviation (s.d.) using GraphPad Prism 6.02 and SPSS 26.0 software; a one-way analysis of variance was used to analyze differences between the two groups, while paired-sample *t* test was used to analyze differences among multiple groups. Statistical significance was defined *p* values < 0.05.

### Supplementary information

24.2.11 Supplementary data

S1

S2

S3

An Author Contribution Statement

AJ-checklist

Original Data File

## Data Availability

The published article includes all data sets generated/analyzed for this study.
