# Supplementary material for: Metabolic remodeling by the PD-L1 inhibitor BMS-202 significantly inhibits cell malignancy in human glioblastoma
Source: Cell Death Dis. 2024 Mar 4;15(3):186. doi: 10.1038/s41419-024-06553-5 (PMC10912212; doi:10.1038/s41419-024-06553-5)
Supplement: Supplementary file 1 — 24.2.11 Supplementary data [file 41419_2024_6553_MOESM1_ESM.doc]

**Figure S1. BMS-202 has an inhibitory activity with PD-L1 expression in GBM cells. A.** Flow cytometry assay of PD-L1 expression on the surface of U251 cells treated with or without 10μM BMS-202. Data from replicates were shown as mean ± s.d. and statistically analyzed using ANOVA, which were shown as barplots with individual points. **B.** The RT-PCR results of PD-L1 in U251 cells transfected with or without siPD-L1-2, together with or without BMS-202, taking β-actin as the internal control. The relative mRNA levels of PD-L1 from replicates in each group were graphically shown as barplots and statistically analyzed using ANOVA. **C.** The western blotting results showing the expression of PD-L1 and β-actin in U251 cells treated with or without indicated concentrations of BMS-202. The relative protein levels of PD-L1 which were normalized to β-actin from replicates were shown as mean ± s.d. and statistically analyzed using ANOVA. **D.** The cell invasion analysis of U251 cells transfected with or without siPD-L1-2 and treated with or without BMS-202 for 24 h. **E.** Triplicated western blotting results showing the knockdown efficiency of siPD-L1-1, -2, -3 in U251 cells, taking siCtrl as a control. *p<0.05, **p<0.01, ***p<0.001.

**Figure S2. BMS-202 treatment causes differential genes expression involving in metabolic progress. A.** Volcano plot showing the differential expression of genes in U251 cells treated with BMS-202, taking the vehicle treated group as a control. Red points indicated upregulated genes,while blue ones indicated downregulated genes. Grey plots indicated genes without significance. **B.** GO Analysis of above differentially expressed genes in U251 cells. The horizontal axis represents different modules, the vertical axis represents the percentage of genes. **C.** KEGG bubble plot. The horizontal axis represents rich factor and the vertical axis represents significant difference. **D.** The detailed metabolism-related processes associated with BMS-202 treatment, including nucleotide biosynthetic process (17.73%), phosphate metabolic process (12.73%), and serine biosynthesis.

**Figure S3.** **A-D.** BMS-202 metabolism-related hub genes, PRPS2 (A), PLCE1 (B), DTYMK (C) and FH (D), are closely associated with glioma poor prognosis.

**Table S1. The top differentially expressed genes in response to BMS-202 treatment in U251 cells.**

| **Gene ID** | **Gene Symbol** | **Type** | **log2 (drug/control)** | **Qvalue**  **(control-vs-drug)** |
| --- | --- | --- | --- | --- |
| 284348 | *LYPD5* | mRNA | 2.428443794 | 0.028039006 |
| 4312 | *MMP1* | mRNA | 1.510631484 | 0.006626632 |
| 102724594 | *U2AF1L5* | mRNA | –2.44916194 | 0.001910273 |
| 4916 | *NTRK3* | mRNA | –1.91823035 | 2.98E-04 |
| 29103 | *DNAJC15* | mRNA | –1.898891187 | 5.75E-04 |
| 401024 | *FSIP2* | mRNA | –1.880571413 | 4.65E-09 |
| 3860 | *KRT13* | mRNA | –1.738015994 | 6.65E-04 |
| 3757 | *KCNH2* | mRNA | –1.40262022 | 0.017283771 |
| 771 | *CA12* | mRNA | 1.423229368 | 0.004067336 |
| 219699 | *UNC5B* | mRNA | 1.380662796 | 0.001707282 |
| 1846 | *DUSP4* | mRNA | 1.350564288 | 1.47E-06 |
| 51296 | *SLC15A3* | mRNA | –1.372319472 | 6.34E-04 |
| 23462 | *HEY1* | mRNA | 1.32869439 | 1.81E-18 |
| 387914 | *SHISA2* | mRNA | 1.322671249 | 0.00103026 |
| 1755 | *DMBT1* | mRNA | 1.302324169 | 0.010308253 |
| 3491 | *CYR61* | mRNA | –1.327144852 | 1.36E-95 |
| 1848 | *DUSP6* | mRNA | 1.22172572 | 0.003614491 |
| 51700 | *CYB5R2* | mRNA | 1.202838753 | 0.018604691 |
| 55879 | *GABRQ* | mRNA | –1.228465102 | 2.39E-05 |
| 167681 | *PRSS35* | mRNA | –1.213059145 | 2.55E-20 |
| 399694 | *SHC4* | mRNA | 1.184437163 | 5.01E-05 |
| 7294 | *TXK* | mRNA | –1.195230737 | 0.020720403 |
| 3434 | *IFIT1* | mRNA | –1.126708042 | 0.012375573 |
| 9518 | *GDF15* | mRNA | 1.138426766 | 0.00386976 |
| 153830 | *RNF145* | mRNA | 1.126457707 | 7.45E-118 |
| 57165 | *GJC2* | mRNA | –1.125946136 | 0.025953821 |
| 4886 | *NPY1R* | mRNA | –1.112228719 | 0.001189163 |
| 445329 | *SULT1A4* | mRNA | 1.06983566 | 4.34E-04 |
| 2119 | *ETV5* | mRNA | 1.056508757 | 3.45E-04 |
| 51200 | *CPA4* | mRNA | –1.069709941 | 1.48E-19 |
| 9311 | *ASIC3* | mRNA | 1.052036801 | 0.01573643 |
| 158376 | *SPAAR* | mRNA | –1.053887726 | 9.49E-04 |
| 10253 | *SPRY2* | mRNA | 1.041379774 | 1.23E-06 |
| 10346 | *TRIM22* | mRNA | –1.035251194 | 0.005444217 |
| 9750 | *RIPOR2* | mRNA | –1.026414755 | 0.030114842 |
| 118738 | *ZNF488* | mRNA | –1.013456203 | 0.00215638 |
| 59277 | *NTN4* | mRNA | –1.011045813 | 3.11E-04 |
| 22861 | *NLRP1* | mRNA | 1.035875734 | 0.012276264 |
| 2118 | *ETV4* | mRNA | 1.031393689 | 6.13E-04 |
| 27063 | *ANKRD1* | mRNA | –1.000796831 | 3.35E-23 |
| 79888 | *LPCAT1* | mRNA | 1.026212059 | 8.10E-72 |

FC represents the ratios between the medication and control groups; log2FC > 0 indicates upregulation and log2FC < 0 indicates downregulation.

**Table S2. Metabolism enrichment of differential genes in response to BMS-202.**

| **Gene Symbol** | **log2 (drug/control)** | **Qvalue**  **(control–vs–drug)** | **Running ES** | **Core Enrichment** | |
| --- | --- | --- | --- | --- | --- |
| PLCE1 | –0.618238174 | 1.39E–10 | –0.003915933 | Yes | |
| RRM1 | –0.330932982 | 1.70E–09 | –0.011493783 | Yes | |
| TKT | –0.317503166 | 0.005176253 | –0.019659318 | Yes | |
| GSTM2 | –0.310804607 | 0.047775788 | –0.026038172 | Yes | |
| SORD | –0.296202942 | 0.003429702 | –0.037870176 | Yes | |
| PRPS2 | –0.266587599 | 0.021663271 | –0.051408403 | Yes | |
| IVD | –0.260498018 | 0.001743666 | –0.06275314 | Yes | |
| MTHFD1 | –0.256747286 | 1.01E–06 | –0.06995916 | Yes | |
| DNMT1 | –0.244449876 | 2.94E–06 | –0.0786564 | Yes | |
| LDHB | –0.236486764 | 7.93E–07 | –0.09094233 | Yes | |
| DTYMK | –0.233076105 | 0.024205522 | –0.10264584 | Yes | |
| FH | –0.227895573 | 0.001212357 | –0.11347957 | Yes | |
| ASL | –0.224556982 | 0.016891078 | –0.12626135 | Yes | |
| CTPS1 | –0.224486882 | 0.009275477 | –0.13691823 | Yes | |
| COX7B | –0.224394121 | 0.005911554 | –0.13840126 | Yes | |
| AHCY | –0.220917659 | 2.23E–04 | –0.14954545 | Yes | |
| ADCY3 | –0.207052882 | 0.033505117 | –0.16113964 | Yes | |
| PGAM1 | –0.193959035 | 1.34E–06 | –0.170907 | Yes | |
| PFKL | –0.192457477 | 0.011617252 | –0.1778962 | Yes | |
| TPI1 | –0.19178701 | 1.88E–07 | –0.18779337 | Yes | |
| PGK1 | –0.189824849 | 5.53E–06 | –0.18576644 | Yes | |
| AGPS | –0.189356202 | 0.002633702 | –0.1957237 | Yes | |
| ALDH7A1 | –0.18924918 | 0.022894743 | –0.20518076 | Yes | |
| PRDX6 | –0.188273076 | 5.41E–04 | –0.21587008 | Yes | |
| COX7C | –0.188235517 | 0.013893384 | –0.22670862 | Yes | |
| ENO1 | –0.183642811 | 2.36E–07 | –0.23723376 | Yes | |
| GLO1 | –0.181589445 | 3.30E–04 | –0.24469504 | Yes | |
| PTGES3 | –0.179453824 | 2.72E–04 | –0.2532871 | Yes | |
| NT5C2 | –0.17823614 | 0.017044774 | –0.2617028 | Yes | |
| PFKP | –0.176466886 | 0.001582583 | –0.2717698 | Yes | |
| GRHPR | –0.172321307 | 0.025899732 | –0.27837425 | Yes | |
| IMPDH2 | –0.169939013 | 0.01061862 | –0.28846657 | Yes | |
| AMD1 | –0.155110817 | 0.01969206 | –0.29571113 | Yes | |
| ATP6V0E2 | –0.141545838 | 0.034683359 | –0.30482632 | Yes | |
| HACD3 | –0.139369737 | 0.019728043 | –0.3132694 | Yes | |
| GPX4 | –0.1320666 | 0.038850245 | –0.3190053 | Yes | |
| PKM | –0.084729704 | 0.031244369 | –0.32549897 | Yes | |
| ATP6V1B2 | 0.139476135 | 0.034303331 | –0.33481357 | Yes | |
| PGD | 0.142815117 | 0.046787841 | –0.34399775 | Yes | |
| ADPGK | 0.161526272 | 0.047404222 | –0.35192955 | Yes | |
| EPRS | 0.163461403 | 6.28E–04 | –0.35565627 | Yes | |
| AHCYL2 | 0.168626636 | 0.049781315 | –0.36186996 | Yes | |
| HSD17B12 | 0.175472196 | 0.002780513 | –0.36659834 | Yes | |
| AGPAT5 | 0.181180959 | 0.029255851 | –0.36943656 | Yes | |
| ENO2 | 0.191836654 | 0.042114233 | –0.37628525 | Yes | |
| STT3A | 0.192753088 | 9.07E–05 | –0.36996436 | Yes | |
| MAN2A1 | 0.193567157 | 0.001397796 | –0.37731192 | Yes | |
| ATP6V0A1 | 0.194708553 | 6.13E–04 | –0.37979466 | Yes | |
| GNPDA1 | 0.196742047 | 0.00439742 | –0.36678678 | Yes | |
| AMPD2 | 0.199526024 | 0.038522205 | –0.3741508 | Yes | |
| PGM3 | 0.220411347 | 0.001645126 | –0.38099945 | Yes | |
| PHGDH | 0.237732723 | 2.69E–12 | –0.3836056 | Yes | |
| TK2 | 0.240926931 | 0.010635336 | –0.378503 | Yes | |
| AKR1A1 | 0.25840509 | 0.001520713 | –0.3829181 | Yes |  |
| PI4K2A | 0.277214848 | 7.05E–05 | –0.3858017 | Yes |  |
| UGDH | 0.27749704 | 6.44E–04 | –0.39178318 | Yes |  |
| ALG2 | 0.287145216 | 0.022996135 | –0.39764148 | Yes |  |
| GPAM | 0.295366557 | 0.011008691 | –0.39996445 | Yes |  |
| UGT8 | 0.297126744 | 0.02652175 | –0.40497556 | Yes |  |
| KDSR | 0.304476569 | 0.008941978 | –0.40519327 | Yes |  |
| GPT2 | 0.313340286 | 5.15E–04 | –0.41019166 | Yes |  |
| ARL1 | 0.317919708 | 4.07E–06 | –0.4146814 | Yes |  |
| PAPSS2 | 0.32544852 | 0.031442351 | –0.42061475 | Yes |  |
| MVD | 0.32655278 | 0.008463439 | –0.4261828 | Yes |  |
| GARS | 0.378500798 | 1.03E–10 | –0.42596778 | Yes |  |
| CYP51A1 | 0.378935182 | 2.72E–12 | –0.43110704 | Yes |  |
| GFPT2 | 0.391502371 | 4.68E–04 | –0.43095878 | Yes |  |
| RDH10 | 0.411409068 | 0.047304841 | –0.43519607 | Yes |  |
| PSAT1 | 0.419650521 | 8.53E–12 | –0.43975988 | Yes |  |
| SMOX | 0.470077341 | 2.00E–04 | –0.44531706 | Yes |  |
| HMGCS1 | 0.667104018 | 0.001042634 | –0.44664437 | Yes |  |
| UGCG | 0.705516346 | 9.30E–09 | –0.4488218 | Yes |  |

**Table S3.** Sequence of primers used in the study.

| Product | Forward sequence (5’-3’) | Reverse sequence (5’-3’) |
| --- | --- | --- |
| siCtrl | GAAUUAAUUAAAGAUGGCCCGUUGUACU | UCAUCGAAGUUAUAGGGAUACAUUACGUGAUC |
| siPD-L1 # 1 | GAGGAAGACCUGAAGGUUCAGCAUA | UAUGCUGAACCUUCAGGUCUUCUC |
| siPD-L1 # 2 | CCUACUGGCAUUUGCUGAAC U | AAUGCGUUCAGCAAAUGCCAGUAGG |
| siPD-L1 # 3 | UGAUACACAUUUGGAGGAGACGUAA | UUACGUCUCCUCCAAAUGUGUAUCA |
| AKT1 | CCGGAATTCATGAGCGACGTGGCTATTGTGAAGG | CCGCTCGAGTCAGGCCGTGCCGCTGG |
| *β-actin* | TGGGCATGGAGTCCTGTG | TCTTCATTGTGCTGGGTG |
| *LAT1* | CATTATACAGCGGCCTCTTTG | GGTTGGTCAGCACGTACACC |
| *TFEB* | CCAAGGAGCGGCAGAAGA | CGGATGTAATCCACAGAGGC |
| *BCAT1* | CAACCCAAAGTTCGTCAG | CGTGCCCACTTCAGTTAT |
| *BCAT2* | CCGCTGAATGGTGTTATCC | CCCGAGCCAAAGACTTCC |
| *CREB1* | TCCGTCTAATGAAGAACAGG | TGATTTGTGGCAGTAAAGGT |
| *PD-L1* | AGAACTACCTCTGGCACAT | ATCCATCATTCTCCCTTT |

**Table S4. A**ntibodies used in the study.

| Protein name | Source | Category no. |
| --- | --- | --- |
| N-caderin | Cell Signaling Technology | 13116T |
| E-caderin | Cell Signaling Technology | 14472S |
| MMP9 | Cell Signaling Technology | 13667T |
| β-catenin | Proteintech | 8480T |
| β-actin | Proteintech | 81115-1-RR |
| IgG | Beyotime | P2177S |
| pAkt(S473) | Cell Signaling Technology | 4060T |
| pAkt(T308) | Cell Signaling Technology | 13038T |
| AKT | Cell Signaling Technology | 4060T |
| GAPDH | Proteintech | 60004-1-Ig |
| PD-L1 | Cell Signaling Technology | 13684T |
| mTOR | Proteintech | 20657-1-AP |
| p-mTOR | Cell Signaling Technology | 5536T |
| P70S6K1 | Proteintech | 14485-1-AP |
| p-P70S6K1 | Abcam | ab32359 |
| BCAT1 | Proteintech | 13640-1-AP |
| BCAT2 | Proteintech | 16417-1-AP |
