# Supplementary figures and images for: Metabolic remodeling by the PD-L1 inhibitor BMS-202 significantly inhibits cell malignancy in human glioblastoma
Source: Cell Death Dis. 2024 Mar 4;15(3):186. doi: 10.1038/s41419-024-06553-5 (PMC10912212; doi:10.1038/s41419-024-06553-5)

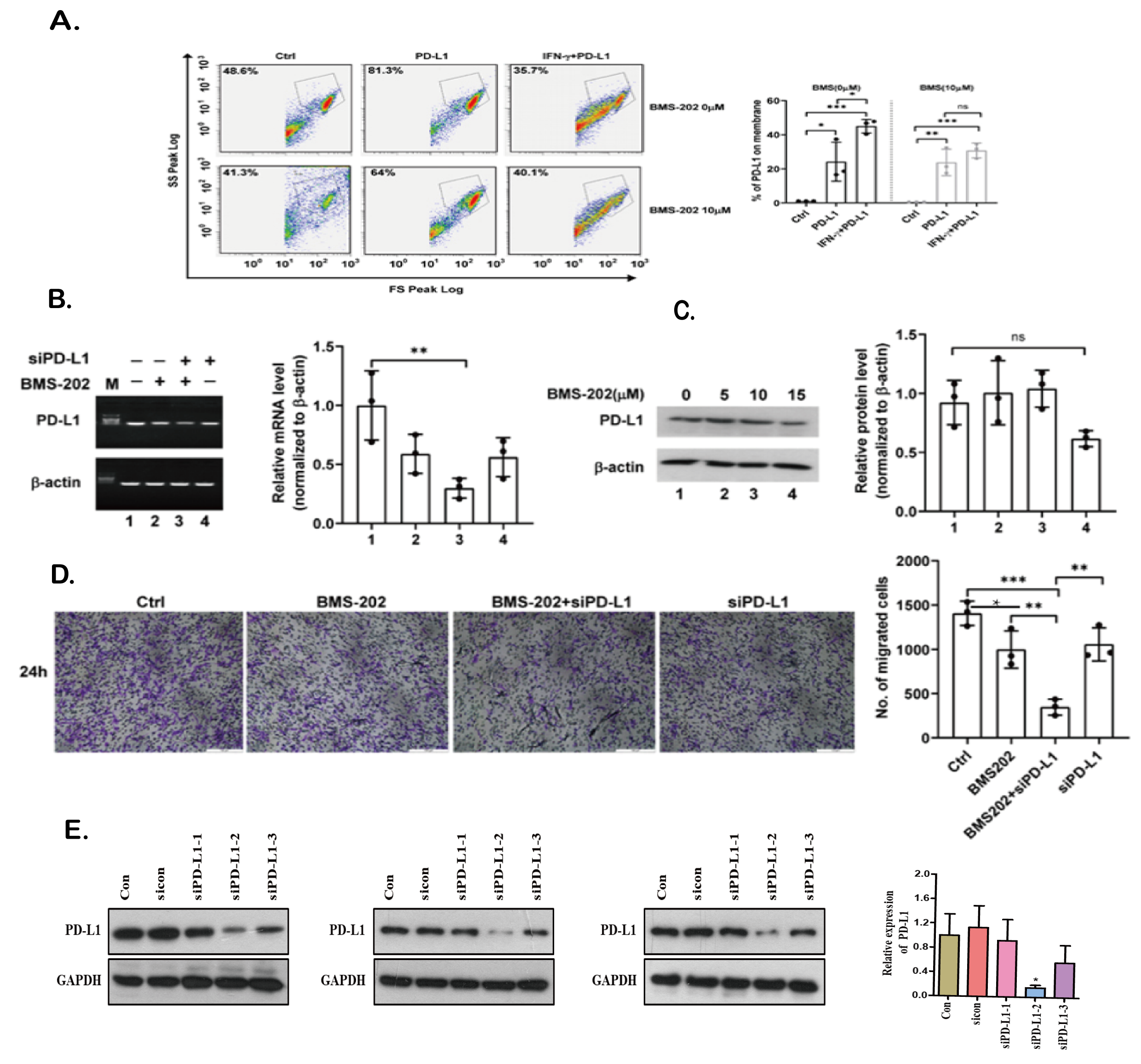

Supplement: Supplementary file 2 — S1 [file 41419_2024_6553_MOESM2_ESM.tif]

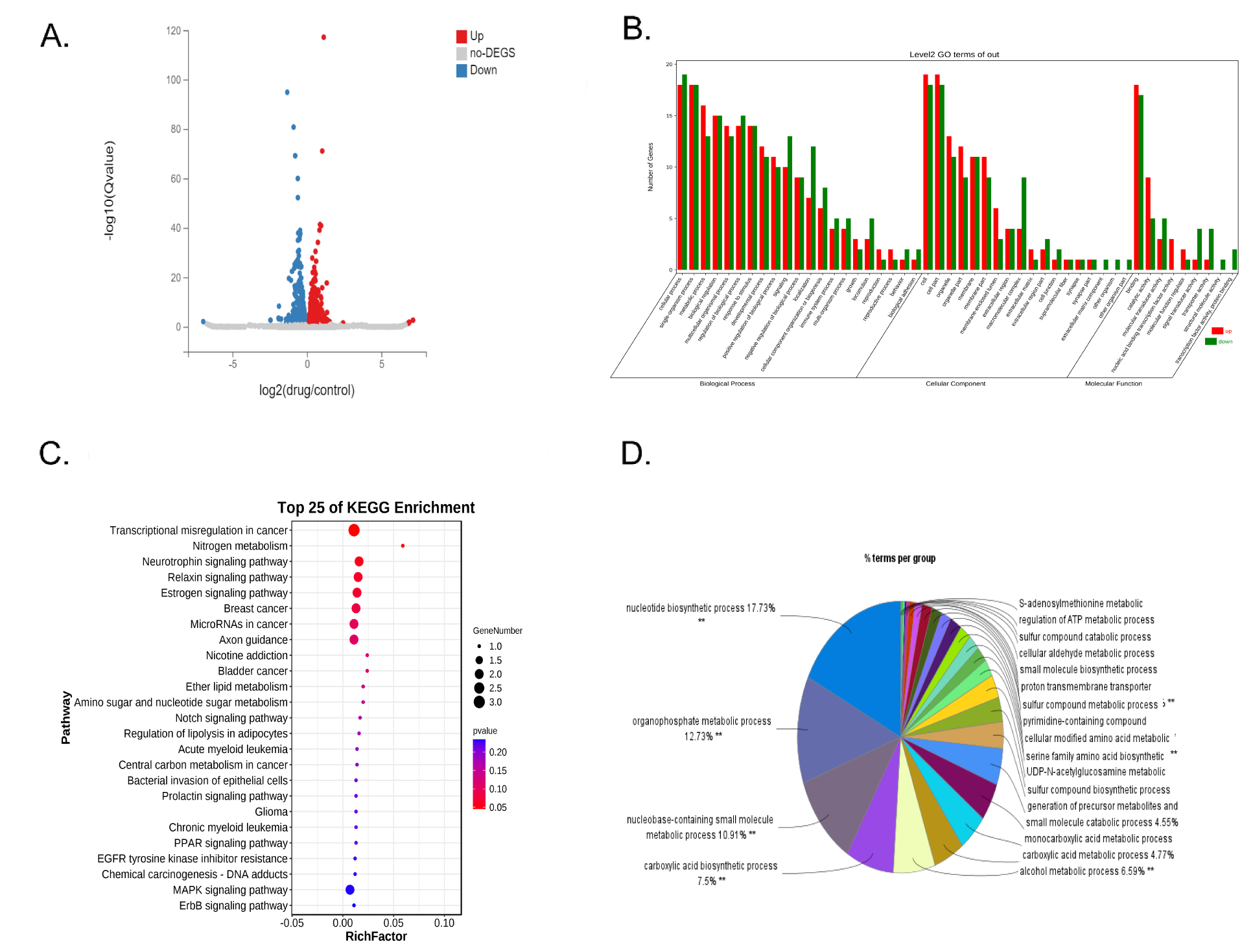

Supplement: Supplementary file 3 — S2 [file 41419_2024_6553_MOESM3_ESM.tif]

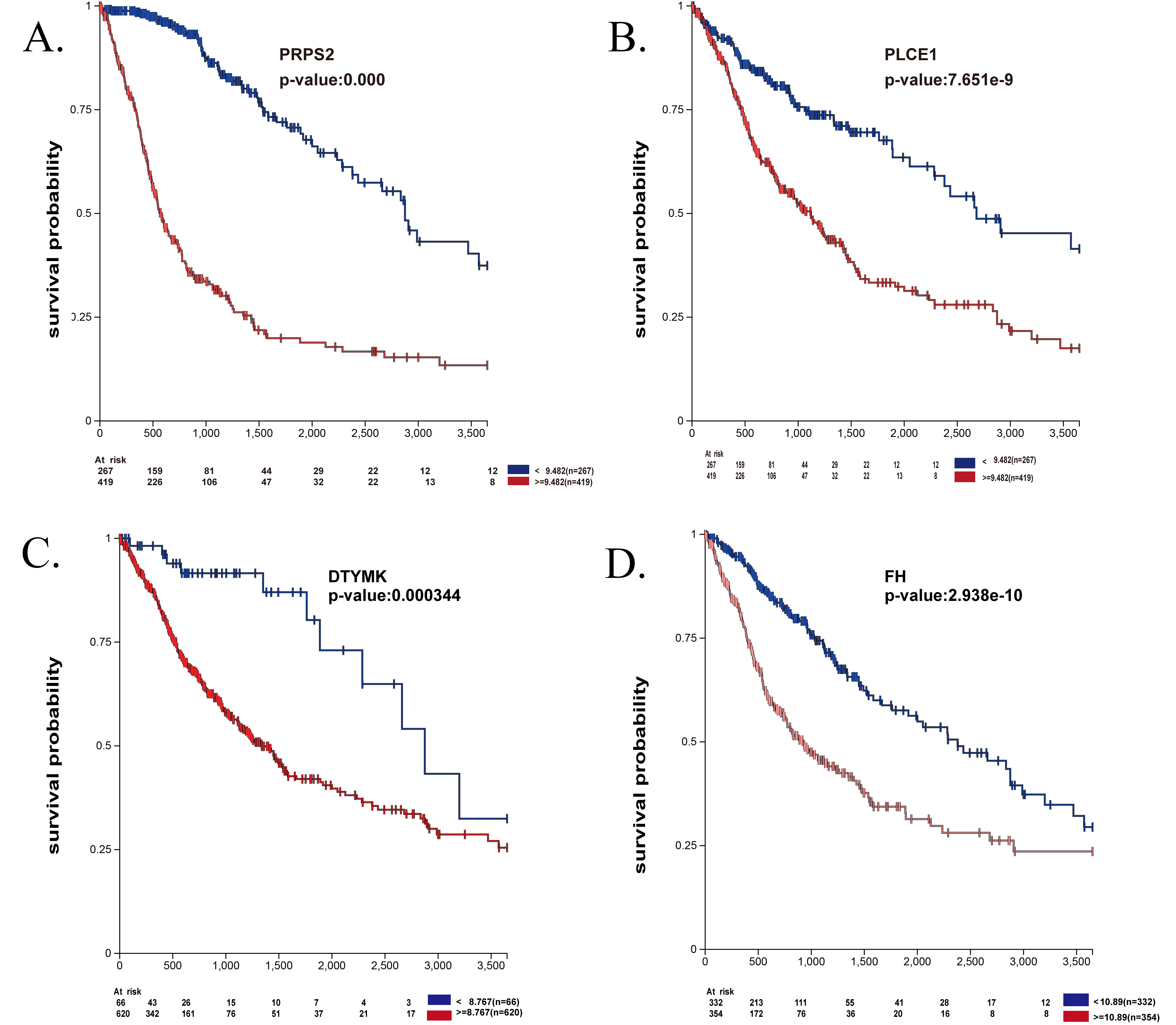

Supplement: Supplementary file 4 — S3 [file 41419_2024_6553_MOESM4_ESM.tif]

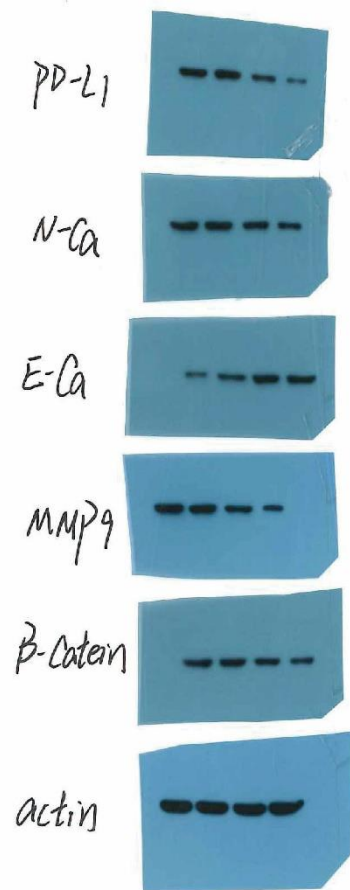

Figure 1H and Figure 2C.

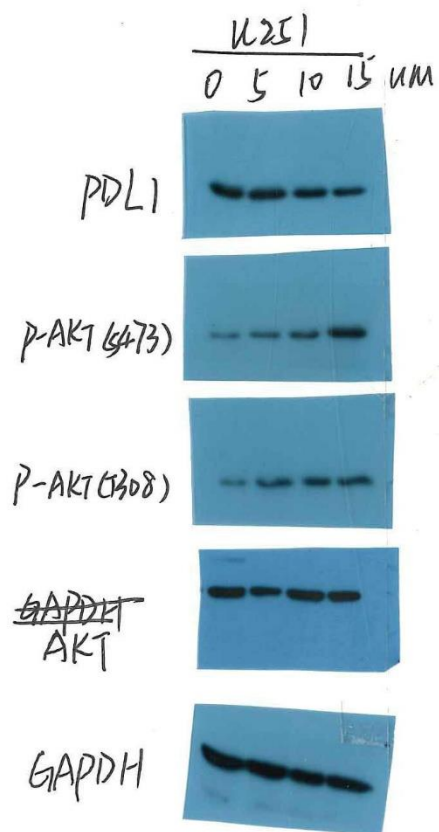

Figure 5A

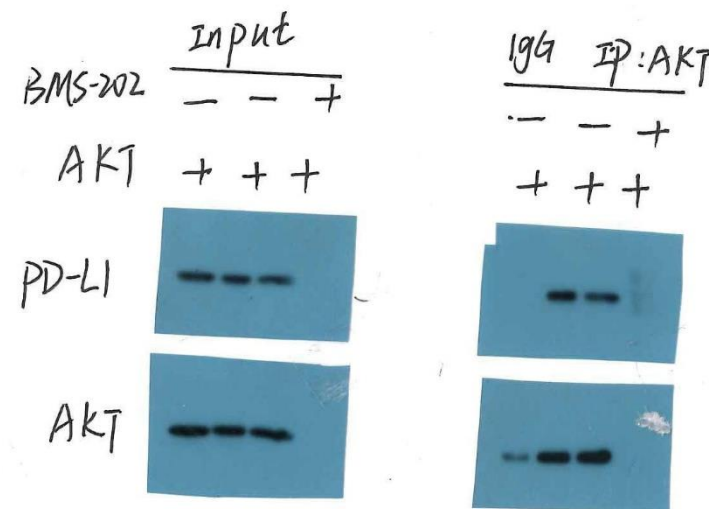

Figure 5B

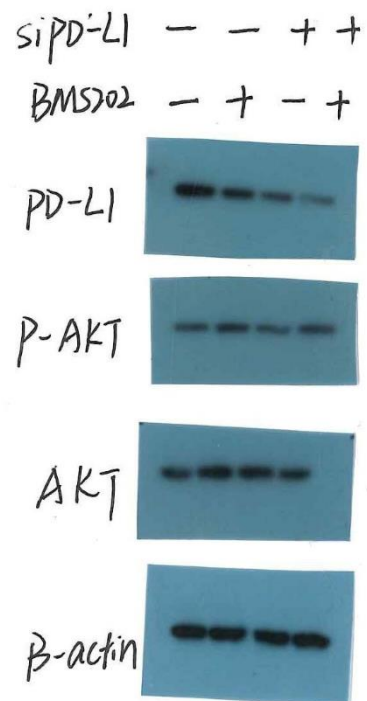

Figure 5C

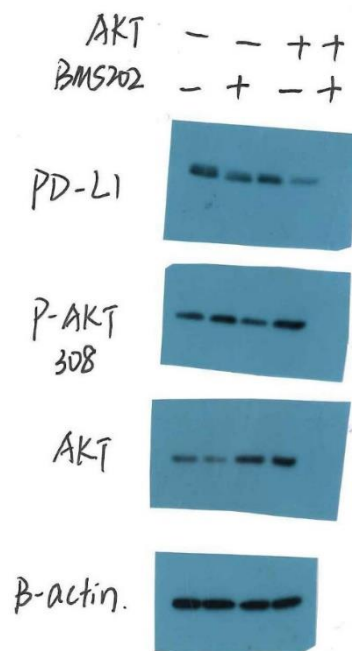

Figure 5D

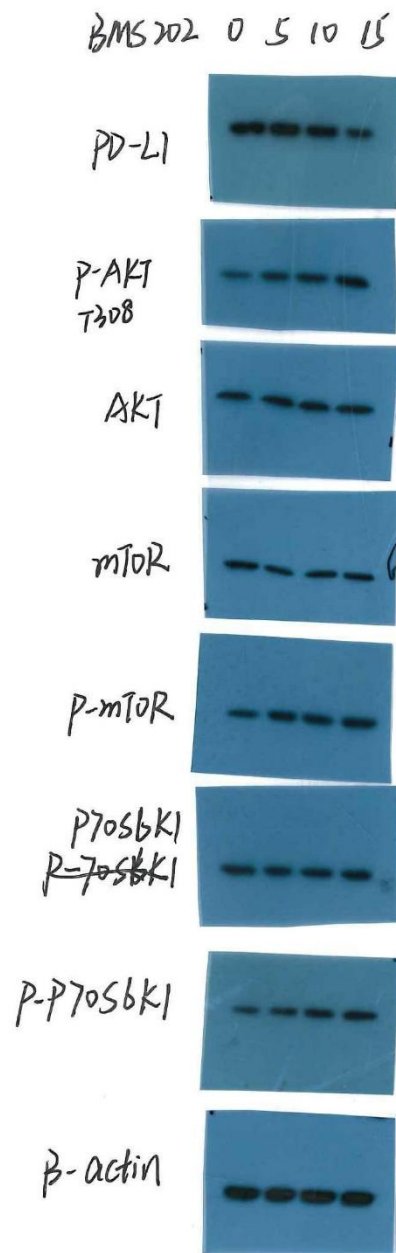

Figure 6A

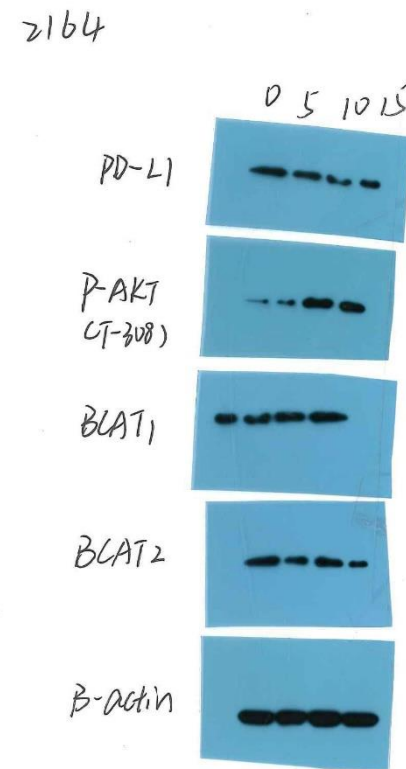

Figure 7A

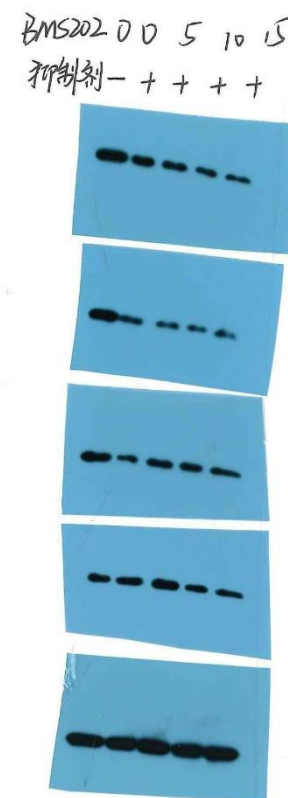

Figure 7B

Supplement: Supplementary file 7 — Original Data File [file 41419_2024_6553_MOESM7_ESM.pdf]
