# Supplementary material for: Metabolic remodeling by the PD-L1 inhibitor BMS-202 significantly inhibits cell malignancy in human glioblastoma
Source: Cell Death Dis. 2024 Mar 4;15(3):186. doi: 10.1038/s41419-024-06553-5 (PMC10912212; doi:10.1038/s41419-024-06553-5)
Supplement: Supplementary file 5 — An Author Contribution Statement [file 41419_2024_6553_MOESM5_ESM.pdf]

YXO, JTH conceived and designed the experiments; YXO, W WJ performed the experiments; YXO, WWJ analyzed the data and drawed the figures; JTH provided the Funding; YXO, WWJ wrote the manuscript; All authors read, verified the underlying data and approved the manuscript.
